# Recurrent *Scedosporium apiospermum* mycetoma successfully treated by surgical excision and terbinafine treatment: a case report and review of the literature

**DOI:** 10.1186/s12941-017-0195-z

**Published:** 2017-04-14

**Authors:** Eszter J. Tóth, Géza R. Nagy, Mónika Homa, Marianna Ábrók, Ildikó É. Kiss, Gábor Nagy, Zsuzsanna Bata-Csörgő, Lajos Kemény, Edit Urbán, Csaba Vágvölgyi, Tamás Papp

**Affiliations:** 1 MTA-SZTE “Lendület” Fungal Pathogenicity Mechanisms Research Group, Hungarian Academy of Sciences, University of Szeged, Közép fasor 52, Szeged, 6726 Hungary; 2 Department of Microbiology, Faculty of Science and Informatics, University of Szeged, Közép fasor 52, Szeged, 6726 Hungary; 3 Department of Dermatology and Allergology, Albert Szent-Györgyi Medical Centre, University of Szeged, Szeged, Hungary; 4 Institute of Clinical Microbiology, Albert Szent-Györgyi Medical Centre, University of Szeged, Szeged, Hungary

**Keywords:** Filamentous fungi, Cutaneous infection, Antifungal susceptibility, Immunosuppression, Corticosteroid therapy, *Scedosporium apiospermum*

## Abstract

**Background:**

*Scedosporium apiospermum* is an emerging opportunistic filamentous fungus, which is notorious for its high levels of antifungal-resistance. It is able to cause localized cutaneous or subcutaneous infections in both immunocompromised and immunocompetent persons, pulmonary infections in patients with predisposing pulmonary diseases and invasive mycoses in immunocompromised patients. Subcutaneous infections caused by this fungus frequently show chronic mycetomatous manifestation.

**Case report:**

We report the case of a 70-year-old immunocompromised man, who developed a fungal mycetomatous infection on his right leg. There was no history of trauma; the aetiological agent was identified by microscopic examination and ITS sequencing. This is the second reported case of *S. apiospermum* subcutaneous infections in Hungary, which was successfully treated by surgical excision and terbinafine treatment. After 7 months, the patient remained asymptomatic. Considering the antifungal susceptibility and increasing incidence of the fungus, *Scedosporium* related subcutaneous infections reported in the past quarter of century in European countries were also reviewed.

**Conclusions:**

Corticosteroid treatment represents a serious risk factor of *S. apiospermum* infections, especially if the patient get in touch with manure-enriched or polluted soil or water. Such infections have emerged several times in European countries in the past decades. The presented data suggest that besides the commonly applied voriconazole, terbinafine may be an alternative for the therapy of mycetomatous *Scedosporium* infections.

## Background

*Scedosporium apiospermum* is a ubiquitous, saprophytic filamentous fungus, which can be isolated mainly from environments affected by human activity (i.e. manure-enriched or polluted soils and water) [1]. It is an emerging opportunist that can cause trauma-associated, localized infections, colonization of pulmonary cavities in patients with predisposing pulmonary disorders (such as cystic fibrosis) and systemic invasive diseases in immunocompromised patients [2]. This mould is notorious for its therapy-refractory nature and resistance for several antifungal agents [1, 3]. Traumatic localized infections can occur in both immunocompromised and immunocompetent persons and are most frequently manifested as mycetomatous, chronic progressive subcutaneous infections, which may also reach the muscles and the bones [1]. *Scedosporium*-caused mycetomas can be found worldwide but most cases have been reported in western Europe, Australia and North America [4–6]. Here, we report a Hungarian patient with recurrent mycetomatous *S. apiospermum* infection successfully treated by surgical excision and terbinafine treatment and review the similar cases reported during the past 2 decades in Europe.

## Case presentation

A 70-year-old man, who worked at a vegetable plantation in previous years, was admitted to our institution with severe edema on the lower right leg and foot, accompanied by pain, fever (38.3 °C), erythema, numerous small-sized papules and abscesses. There was no medical history of notable trauma, however the patient did undergo a prosthesis implantation surgery of the right knee, 10 years ago.

In the previous year, he was hospitalized because of melena and anemia. The patient was given 8 units of red blood cell transfusions and underwent gastroscopy, colonoscopy and abdominal computed tomography angiography. Comprehensive gastrointestinal examination found no source of bleeding, however, laboratory tests showed significant proteinuria (10 g/day), low albumin (22 g/L) and IgG (5.24 g/L) levels. A kidney biopsy was performed, which revealed focal segmental glomerulosclerosis. Owing to the possibility of myeloma multiplex and amyloidosis, additional tests were also carried out with negative results. Based on these findings, the patient was diagnosed with nephrotic syndrome and received corticosteroid pulse therapy (3 × 750 mg) intravenously, which was then switched to oral administration. In the following weeks, the corticosteroid dose was gradually reduced from 80 to 16–8 mg, without any complications.

Three months later, he developed fever (38.3 °C), swelling and erythema on both legs. In addition, small petechiae could also be observed on his arms, torso and right knee, which was interpreted as vasculitis caused by the patient’s septic state. Laboratory results displayed profoundly high C-reactive protein (CRP; 354.9 mg/L) level and erythrocyte sedimentation rate (94 mm/h). Based on the clinical symptoms, erysipelas and sepsis were considered to be the initial diagnoses and the patient was put on a course of antibiotic treatment (3 × 1.2 g amoxicillin and clavulanic acid). After 4 days, subsequent laboratory values for CRP and erythrocyte sedimentation rate showed 76.8 mg/L and 84 mm/h, respectively.

**Fig. 1:**
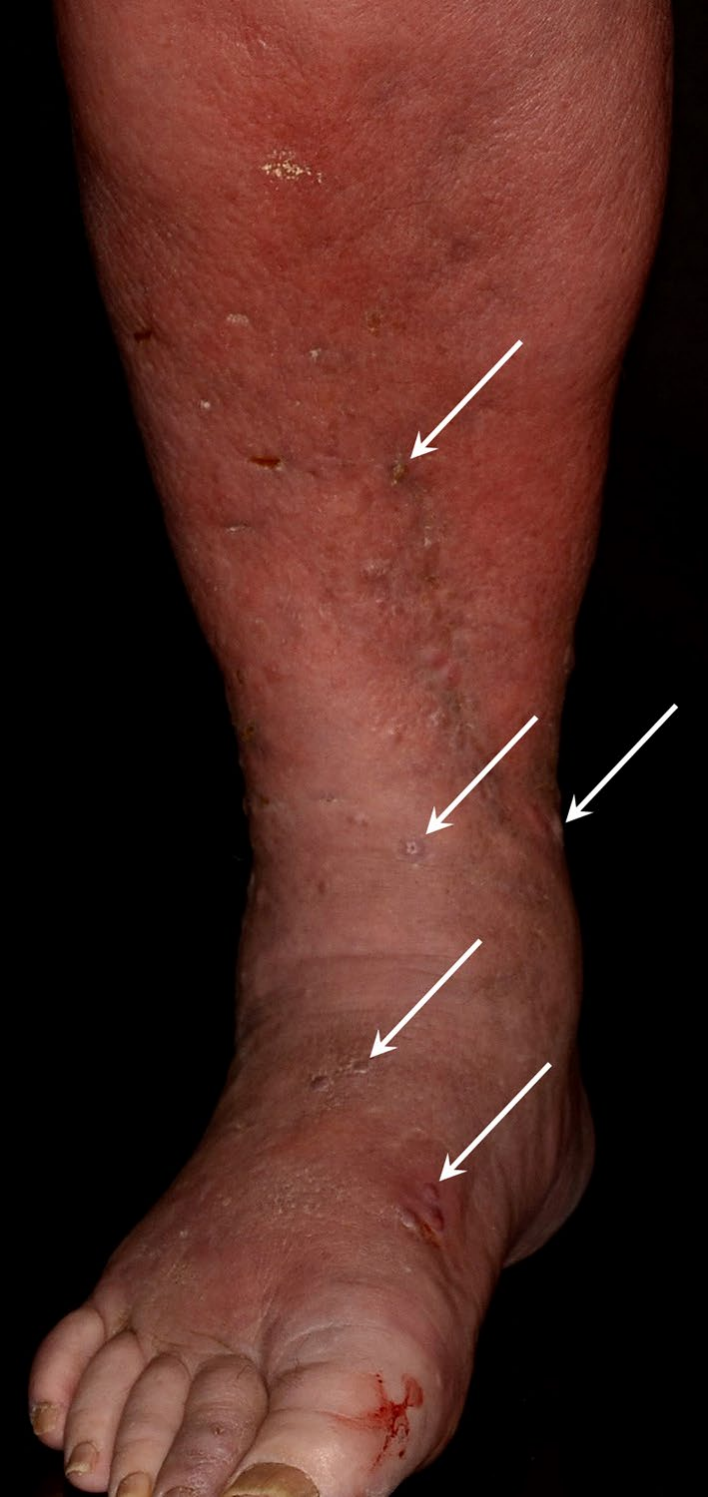
Edema, erythema and small superficial abscesses (*white arrows*) on the lower right leg and foot

After 2 weeks, however, the patient was referred to our clinic, owing to the progression in his cutaneous symptoms. The patient then presented with a severely oedemic and painful lower right leg, with slight erythema and multiple small soft papules (Fig. 1). In addition, the whole skin of this extremity had a sponge-like feel to the touch. No notable symptoms were present on the left leg. Laboratory tests showed elevated CRP (38 mg/L) and high white blood cell count (12.85 × 10^9^/L). Ultrasound revealed four 10–13 mm-sized subcutaneous collections of echogenic fluid in the right leg. After incision and drainage of these lesions, swabs were sent to the microbiological laboratory for culturing. Only *Scedosporium apiospermum* grew in culture after 3 days of incubation at normal atmosphere at 30 °C. After accurate morphological assessment, the sample was sent for additional identification by DNA sequencing.

Considering this finding, instead of the initially administered penicillin-type antibiotic, the patient was given fluconazole (200 mg) intravenously on six occasions, to which his symptoms markedly improved. Subsequently, taking the patient’s low glomerular filtration rate (56 mL/min) into account, he was prescribed a synthetic allylamine antifungal medication (250 mg terbinafine) to take every second day with the aim of minimizing potential side effects and underwent several other abscess drainages in the following weeks, in addition to the local antimicrobial agents (i.e. betadine, hydrogen peroxide, and boric acid powder). Parallel with his treatment for the mycotic infection, his steroid therapy dose was gradually decreased and his nephrotic syndrome improved. Two months into his treatment, he experienced a recurrent infection on the right leg. At this time, no mycotic infection could be proven, as only *Staphylococcus aureus* was cultured from multiple small superficial abscesses, thus the patient received a combination antibiotic of piperacillin and tazobactam (3 × 4.5 g) for a week, followed by amoxicillin and clavulanic acid (4 × 1.2 g) for another week, to which his symptoms regressed.

Though edema of the right leg persisted even after many months, there were no longer any clinical signs of inflammation and the patient did not experience any pain. Leukocyte scintigraphy displayed increased activity around the right ankle, suggesting the development of a new abscess, but it was successfully managed with antibiotic and local treatment. After 7 months, the patient remained asymptomatic and currently maintains a low dose of corticosteroid (4 mg/day) therapy. In addition to occupational exposure, low serum IgG levels and the immunosuppressive effect of the corticosteroid treatment were most likely compelling factors in further substantiating the likelihood of this rare fungal infection in our patient’s case.

## Mycological investigations

**Fig. 2:**
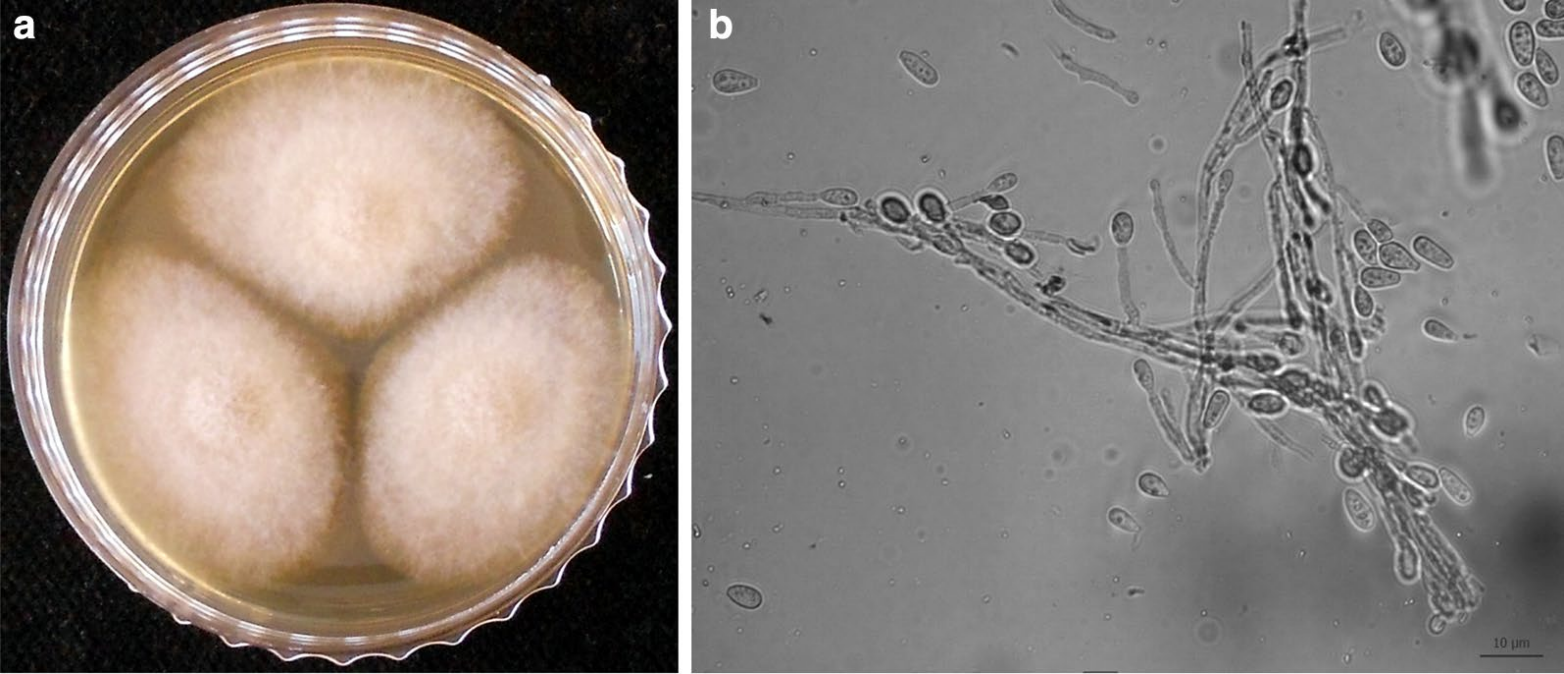
Colony (**a**) and micromorphology (**b**) of *Scedosporium apiospermum* isolate SZMC 23374. *Scale bar* 10 µm

The isolate was deposited in the Szeged Microbiology Collection (SZMC; http://www.wfcc.info/ccinfo/collection/by_id/987) with the strain number SZMC 23374 and was sub-cultured on malt extract agar (MEA; 5% malt extract, 1% glucose, 2% agar) plates at 37 °C for further morphological and molecular examinations. It formed greyish–white, cotton-like colonies on MEA (Fig. 2a). Microscopic examination revealed branched hyphae with clavate or ovoid conidia born singly on simple or branched conidiophores or laterally on the hyphae (Fig. 2b).

For molecular identification, genomic DNA was extracted from 10 mg of mycelium ground to a fine powder in liquid nitrogen and purified by using the MasterPure Yeast DNA purification kit (Epicentre, USA) according to the instructions of the manufacturer. The complete ITS (internal transcribed spacer, including ITS1-5.8S rRNA-ITS2) region was amplified by PCR, using the primers ITS1 and ITS4 [7]. The resulting amplicons were sequenced at LGC Genomics (Germany). The determined sequence was deposited in the European Nucleotide Archive (ENA, http://www.ebi.ac.uk/ena) database (Accession number: LT703449) and used for similarity searches in the NCBI database using the BLASTN algorithm (http://blast.ncbi.nlm.nih.gov/Blast.cgi), which confirmed the fungus as *S. apiospermum* (identities for the whole ITS region, 99%).

In vitro susceptibility of the *S. apiospermum* isolate to amphotericin B (AMB), fluconazole (FLC), itraconazole (ITC), voriconazole (VRC), caspofungin (CSP), natamycin (NTM) and terbinafine (TRB) (Sigma-Aldrich, USA) was tested using the broth microdilution method according to the CLSI guideline M38-A2 [8]. Minimal inhibitory concentration (MIC) values were determined in 96-well microtiter plates by visual inspection of the cultures after 72 h incubation at 37 °C in standard RPMI-1640 medium (Sigma-Aldrich, USA). Inocula were prepared from 14-day cultures grown on MEA; the final conidial suspensions contained 10^4^ CFU/mL. Final concentrations of the drugs ranged from 0.5 to 256 μg/mL. Each plate contained a sterile control (containing medium alone) and a growth control (containing inoculated medium without the drugs). Each experiment was performed in triplicates; *Paecilomyces variotii* ATCC 22319 was used as the quality control strain. Table 1 shows the antifungal susceptibility of the case isolate to the most frequently used antifungal agents. The strain showed low susceptibility to AMB, CSP, ITC and TRB. FLC and NTM proved to be ineffective against the isolate in the investigated concentration range (MIC >128 μg/mL). It was the most susceptible to VRC (MIC 2 μg/mL).

**Table 1:** MIC values (μg/mL) of antifungal drugs to the *S. apiospermum* isolate SZMC 23374

Aetiological agent	MIC (µg/mL)						
	AMB	CSP	FLC	ITC	NTM	TRB	VRC
*S. apiospermum*	16	32	>256	32	>128	64	2

*AMB* amphotericin B, *CSP* caspofungin, *FLC* fluconazole, *ITC* itraconazole, *NTM* natamycin, *TRB* terbinafine, *VRC* voriconazole

## Discussion

Mycetoma (syn. Madura foot) is the infection of the skin and subcutaneous tissues, which usually develops after a traumatic event of the leg or the foot. It is endemic in tropical and subtropical countries, but rarely reported from temperate regions, as well. Based on the causative agents, the disease can be divided into two categories: actinomycetoma or actinomycosis is caused by Actinomycetes, while eumycetoma or maduramycosis is caused by filamentous fungi [2].

*Scedosporium apiospermum* was first isolated in 1909 as the aetiological agent of a white grain mycetoma in Sardinia by Tarozzi [9, 10]. This species was considered to be the anamorph of *Scedosporium boydii* (previously known as *Pseudallescheria boydii*) until 2005, when they proved to be two distinct species based on molecular, physiological and biochemical data [11]. According to the latest molecular studies *S. apiospermum* is a species complex, comprising five distinct species: *S. apiospermum*, *Scedosporium aurantiacum*, *S. boydii*, *Scedosporium minutisporum*, and *Scedosporium dehoogii*. Out of them, *S. apiospermum*, *S. aurantiacum* and *S. boydii*, are regularly isolated from human infections [12]. Their incidence seems to be increasing, especially among immunocompromised patients. After *Aspergillus fumigatus*, *S. apiospermum* and *S. boydii* are the second most frequently isolated moulds in cystic fibrosis patients [13].

**Fig. 3:**
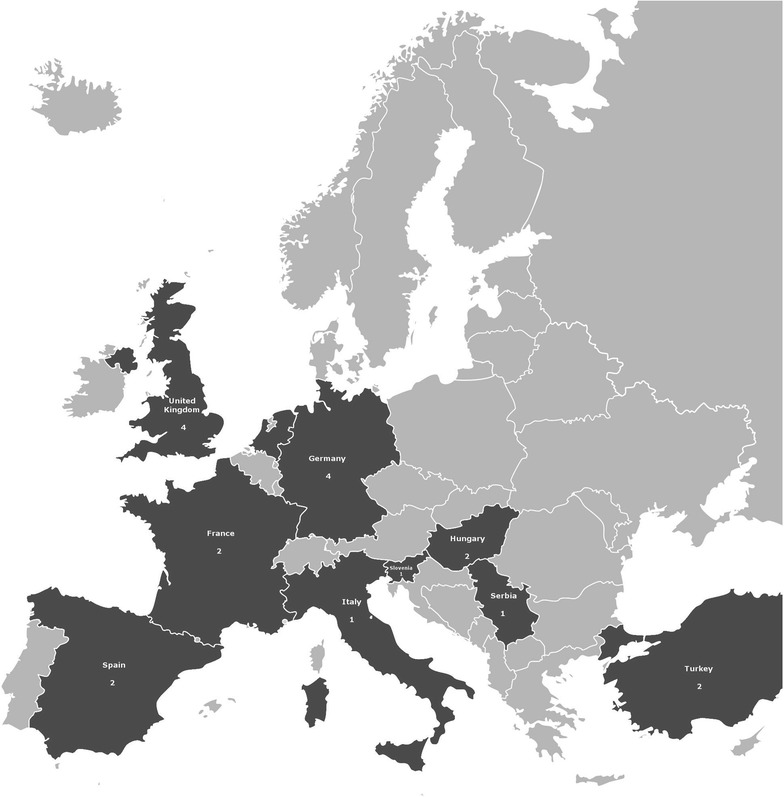
Distribution of subcutaneous *Scedosporium* infections in Europe since 1990

Based on the available case reports in English and Spanish in the PubMed (http://www.ncbi.nlm.nih.gov/pubmed) and Google Scholar (https://scholar.google.hu/) databases, 20 cases of mycetoma or subcutaneous infections were reported due to *Scedosporium* species between 1992 and 2015 from ten European countries: 4–4 from Germany and the UK, 2–2 from The Netherlands, Spain, France, and Turkey and 1–1 from Italy, Serbia, Slovenia, and Hungary [14–32] (Table 2; Fig. 3). Two additional cases have been diagnosed in France, but these patients arrived from outside of Europe (Ivory Coast and Martinique) for hospitalization [33, 34] (not included in Table 2). Of the 20 European cases, 15 were registered in immunosuppressed patients having different underlying conditions, such as organ transplantations, acute myeloid leukemia (AML) or chronic obstructive pulmonary disease (COPD). Similarly to the presented case, out of these patients, 12 had been given a corticosteroid treatment. Although it was not yet proven that these drugs would play a role in the development of *Scedosporium* infections not just as immunosuppressants but as fungal growth stimulators as well, hydrocortisone has previously proven to enhance the growth of Aspergilli [35]. Taking into account that the patient in the present case had worked with manured garden soil during the corticosteroid therapy, characteristic preference of the fungus for such eutrophic and manure-enriched environments [1] could also represent a serious risk factor for the patient besides the corticosteroid treatment.

**Table 2:** Subcutaneous infections caused by *Scedosporium* species in Europe since 1990

Age, gender	Immune status	Predisposing conditions	Signs, symptoms	Aetiological agent	Site of infection	Therapy	Outcome	References
The Netherlands								
59, M	Immunosupp.	Renal transplantation, IV catheter	Purple nodules, slight desquamation	*S. apiospermum*	Left hand and lower arm	VRC	Recovered	[14]
84, M	Immunosupp.	COPD, Periprosthetic fracture	Inflammation with bullae	*S. apiospermum*	Right elbow	VRC	Death after good initial response	[14]
Germany								
24, M	Immunocomp.	Fracture of the tibia	Edematous swellings	*S. boydii, Nocardia abscessus*	Lower left leg	ITC	Recovered	[15]
58, M	Immunosupp.	Chronic glomerulonephritis, Renal transplantation, Insect sting	Painful, swollen induration	*S. apiospermum*	Right forefoot	MCZ, Vacuum seal and suction technique	Recovered	[16]
15, M	Immunosupp.	Lupus erythematodes, Tibia and fibula fracture	Swelling, impaired wound healing	*S. boydii*	Lower leg	VRC	Recovered	[17]
53, F	Immunosupp.	AML	Reddish, inflammatory, painless tumor with central necrosis	*S. apiospermum*	Right forearm	CSP, VRC	Recovered	[18]
Spain								
58, M	Immunosupp.	Chronic glomerulonephritis	Fever, cutaneous lesions	*S. apiospermum*	Both legs	VRC	Recovered	[19]
51, M	Immunosupp.	Renal transplantation	Nodular lesions	*S. apiospermum*	Left heel	ITC, SI	Recovered	[20]
France								
79, M	Immunosupp.	Bronchospasm	Fever, bullous and necrotic purpura	*S. apiospermum*	n.a.	ITC	Death (due to *Pseudomonas* lung infection)	[21]
65, M	Immunosupp.	COPD, IV catheter	Purple nodules, slight desquamation	*S. apiospermum*	Left wrist and lower arm	VRC	Recovered	[22]
UK								
51, M	Immunocomp.	Stepped in a dung fork	Swollen, painful foot	*S. boydii*	Foot (bone involvement)	ITC	Recovered	[23]
71, M	Immunosupp.	AML	Painful, swollen, erythematous foot, necrotic ulcer between toes	*S. apiospermum*	Right foot	ITC	Recovered	[24]
81, M	Immunosupp.	Renal impairment, Pulmonary fibrosis, Scratch from a bush.	Tender subcutaneous nodules	*S. apiospermum*	Left forearm (joint involvement)	ITC	No follow up	[25]
35, M	Immunocomp.	na	Painless swelling	*S. apiospermum*	Right ankle	VRC	Recovered	[26]
Italy								
59, F	Immunosupp.	Renal transplantation	Pain, Achilles tendonitis	*S. apiospermum*	Skin, knee, and Achilles tendon of the left leg	VRC	Recovered	[27]
Serbia								
50, F	Immunocomp.	na	Pain, indurations, local redness	*S. boydii*	Soft tissue and bone involvement	SI	Recovered	[28]
Slovenia								
64, M	Immunosupp.	Microscopic polyangiitis	Swelling, pain	*S. boydii*	Left leg and foot	VRC	Recovered	[29]
Turkey								
48, M	Immunocomp.	na	Swelling	Bacteria, *S. boydii*	Right hand	Amputation	Recovered	[30]
62, F	Immunosupp.	Renal transplantation	Edema, erythema, painful, indurated lesion	*S. boydii*	Left leg	ITC	Recovered	[31]
Hungary								
63, M	Immunosupp.	AML	Swelling, tenderness	*S. apiospermum*	Lower left leg	ITC	Recovered	[32]
70, M	Immunosupp.	Nephrotic syndrome	Pain, edema, fever, erythema	*S. apiospermum*	Lower right leg and foot	TRB, SI	Recovered	Present case

*AML* acute myeloid leukemia, *COPD* chronic obstructive pulmonary disease, *CSP* caspofungin, *F* female, *Immunocomp.* immunocompetent, *Immunosupp.* immunosuppressed, *ITC* itraconazole, *IV* intravenous, *M* male, *MCZ* miconazole, *na* not available, *SI* surgical intervention, *TRB* terbinafine, *VRC* voriconazole

The causative agents were identified as *S. apiospermum* and *S. boydii* in 15 and 7 cases, respectively. While, coinfections of Scedosporia and bacteria (i.e. *Nocardia abscessus* or unidentified Gram+ and Gram− bacteria) were recorded in two cases [15, 30]. However, the real incidence of the two species is unknown, since much of the case reports have been published before 2005 and mention *S. boydii* and *S. apiospermum* as the sexual and asexual form of the same organism. It is also worth to mention that according to a recent case study of Chen et al. [13], the discrimination of *S. apiospermum* and *S. boydii* does not result in a different therapeutic approach.

Sixteen of the cases identified in our literature review had positive outcome, one patient did not show up for follow-up examinations, while in another case, the infected limb has been amputated. In addition to these, two fatal cases have also been recorded, however they could not be connected to the infections caused by Scedosporia. Based on the summarized literature data in Table 2, the most common successfully applied medical therapy involved azole antifungals, i.e. voriconazole or itraconazole. Reimann et al. [16] achieved improvement using miconazole combined with vacuum seal and suction technique. Combination antifungal therapy was reported in one case, when after a failed treatment with fluconazole and liposomal amphotericin B, the patient was cured successfully with the combination of voriconazole and caspofungin [18]. Surgical excision alone without antifungal therapy was applied in one case [28], while amputation without attempting any antifungal treatment was performed once in Turkey [30]. Medical therapy (itraconazole) plus surgery was used in the case reported by Montero et al. [20]. Amphotericin B has been applied several times, but it has always been replaced by other antifungals due to its toxicity or ineffectiveness. Based on these data, the treatment applied in the present case is unique in the European literature, since terbinafine alone or in combination with other antifungals or surgical intervention has not been applied successfully for the treatment of *S. apiospermum* mycetoma before. Although, an *S. apiospermum* skin infection has been successfully treated by terbinafine combined with voriconazole and surgical excision in Spain [36].

We also would like to highlight here that the patient responded well to terbinafine treatment, despite the relatively high in vitro MIC of the drug. A similar phenomenon was observed by Cunningham and Mitchell [24] in case of amphotericin B. These findings underline the need to handle with care the in vitro antifungal susceptibility data in the clinical treatment of filamentous fungi.

## Abbreviations

AMB: amphotericin B
AML: acute myeloid leukemia
COPD: chronic obstructive pulmonary disease
CRP: C-reactive protein
CSP: caspofungin
ENA: European nucleotide archive
FLC: fluconazole
ITC: itraconazole
ITS: internal transcribed spacer
MEA: malt extract agar
MIC: minimal inhibitory concentration
NTM: natamycin
TRB: terbinafine
VRC: voriconazole
SZMC: Szeged microbiology collection
